# Supplementary material for: Compromised transcription-mRNA export factor THOC2 causes R-loop accumulation, DNA damage and adverse neurodevelopment
Source: Nat Commun. 2024 Feb 8;15:1210. doi: 10.1038/s41467-024-45121-5 (PMC10853216; doi:10.1038/s41467-024-45121-5)
Supplement: Supplementary file 1 — Supplementary Information [file 41467_2024_45121_MOESM1_ESM.pdf]

Supplementary Information:

Compromised transcription-mRNA export factor THOC2 causes R-loop accumulation, DNA damage and adverse neurodevelopment

Rudrarup Bhattacharjee[1,3], Lachlan A Jolly[2,3], Mark A Corbett[1,3], Ing Chee Wee[4], Sushma R Rao[1,5], Alison E Gardner[1,3], Tarin Ritchie[1,3], Eline J H van Hugte[6], Ummi Ciptasari[6], Sandra Piltz[2,3,7], Jacqueline E Noll[8], Nazzmer Nazri[1,9], Clare L van Eyk[1,3,] Melissa White[2,3,7], Dani Fornarino[1,3], Cathryn Poulton[10], Gareth Baynam[10,11,12], Lyndsey E Collins-Praino[4], Marten F Snel[1,5], Nael Nadif Kasri[6], Kim M Hemsley[1,9], Paul Q Thomas[2,3,7], Raman Kumar[1,3]†, and Jozef Gecz[1,3]†*

This **Supplementary Information** file contains**:**

1. Supplementary Figures 1 to 8
2. Gating Strategy for Flow Cytometry (Supplementary Fig. 9)

Supplementary Fig. 1

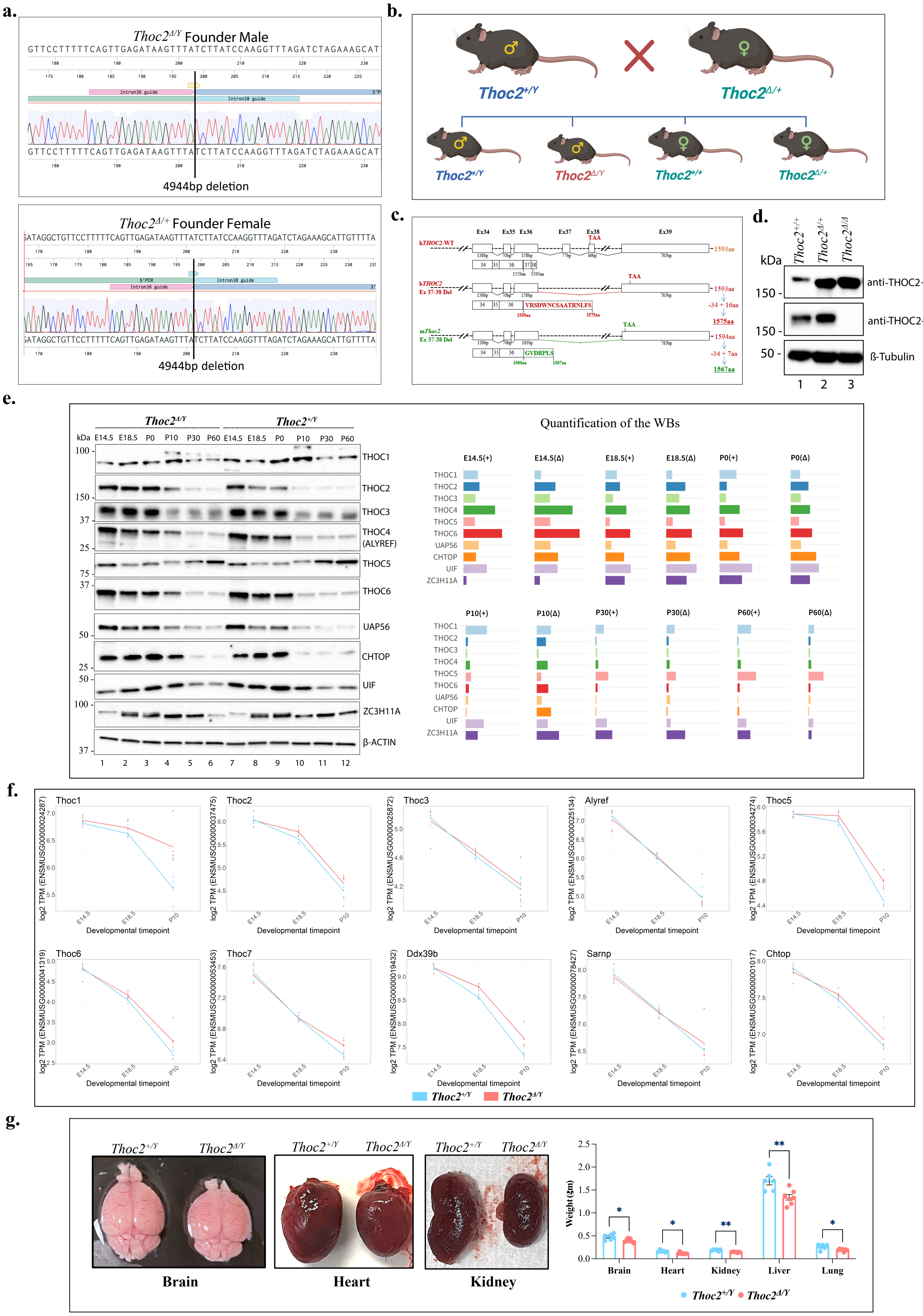

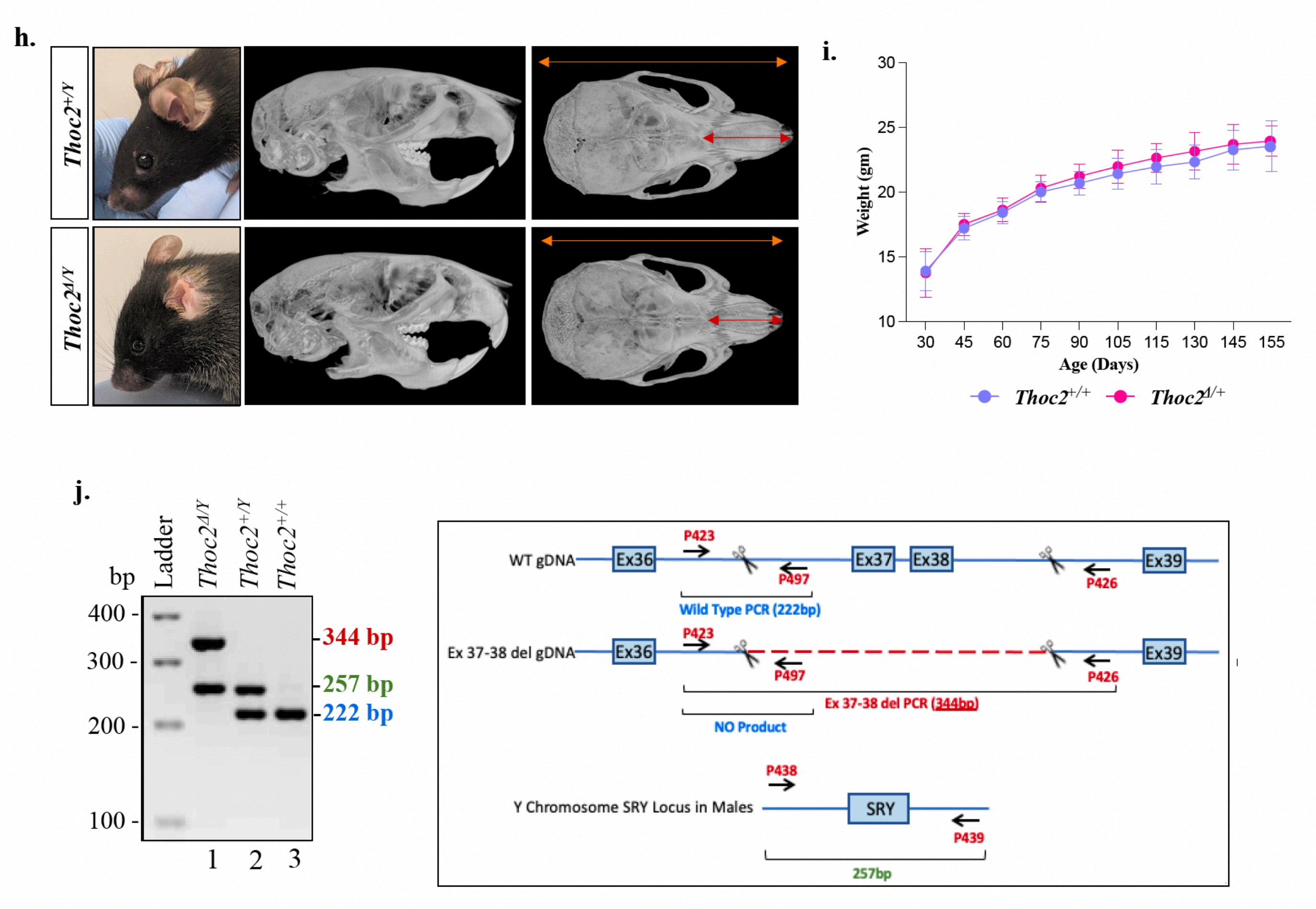

Supplementary Fig. 1: Characterization of *Thoc2*$^{\Delta/Y}$ mice. a, Representative Sanger sequencing chromatograms of *Thoc2* DNA amplified from *Thoc2*$^{\Delta/Y}$ and *Thoc2*$^{\Delta/+}$ founder mice confirming deletion of exon 37 and 38 (4944 deletion). **b,** Schematic presentation of breeding strategy to generate *Thoc2*$^{+/Y}$, *Thoc2*$^{\Delta/Y}$ and *Thoc2*$^{\Delta/+}$mice. **c,** Schematic showing amino acid sequence of C-terminally truncated THOC2 protein in human and mouse. **d,** Western blot showing THOC2 protein in *Thoc2*$^{+/+}$, *Thoc2*$^{\Delta/+}$, and *Thoc2*$^{\Delta/\Delta}$ female mouse brains. Total brain protein lysates were western blotted using anti-THOC2-I antibody (epitope located between amino acids 1097-1200) and anti-THOC2-II antibody (epitope located between amino acids 1543–1593). **e,** Western blot showing different TREX subunit proteins in *Thoc2*$^{+/Y}$ and *Thoc2*$^{\Delta/Y}$ mouse brains at embryonic day E14.5, E18.5 and postnatal day P0, P10, P30 and P60. Right panel: Bar graphs showing levels of TREX subunit proteins quantified from the blot in the left panel. **f,** Graphs showing mRNA expression of TREX subunits in *Thoc2*$^{+/Y}$ and *Thoc2*$^{\Delta/Y}$ mouse brains at embryonic day E14.5, E18.5 and postnatal day P10 (this paper). **g,** Representative images of brain, heart, and kidney from *Thoc2*$^{+/Y}$ and *Thoc2*$^{\Delta/Y}$ mice. Graph showing comparison of weight of the organs ($n \geq 7$ mice per genotype). Data are presented as mean values ± SEM; *$p < 0.05$; **$p < 0.01$; two-way ANOVA, Bonferroni's multiple comparison test. **h,** Representative images and microCT scans of *Thoc2*$^{+/Y}$ and *Thoc2*$^{\Delta/Y}$ mice head showing their facial features (left) and skeletal structure (right). **i,** Graph showing comparison of weight of *Thoc2*$^{+/+}$ and *Thoc2*$^{\Delta/+}$ mice (n= 19 mice per genotype). Data are presented as mean values ± SEM. **j,** Left Panel: Representative genotyping agarose gel image. Lane 1: *Thoc2*$^{\Delta/Y}$ genotype; Lane 2: *Thoc2*$^{+/Y}$ genotype; Lane 3: *Thoc2*$^{+/+}$ female genotype. 344 bp band is exon 37-38 deletion specific; 257 bp band is male SRY specific; 222 bp band is normal *Thoc2* allele specific. Right panel: Schematic presentation of genotyping strategy for the *Thoc2* exon 37-38 deletion mouse model. Locations of primers and sizes of the expected PCR amplified products are shown. Source data are provided as a Source Data file. Fig. 1b was created with BioRender.com.

Supplementary Fig. 2

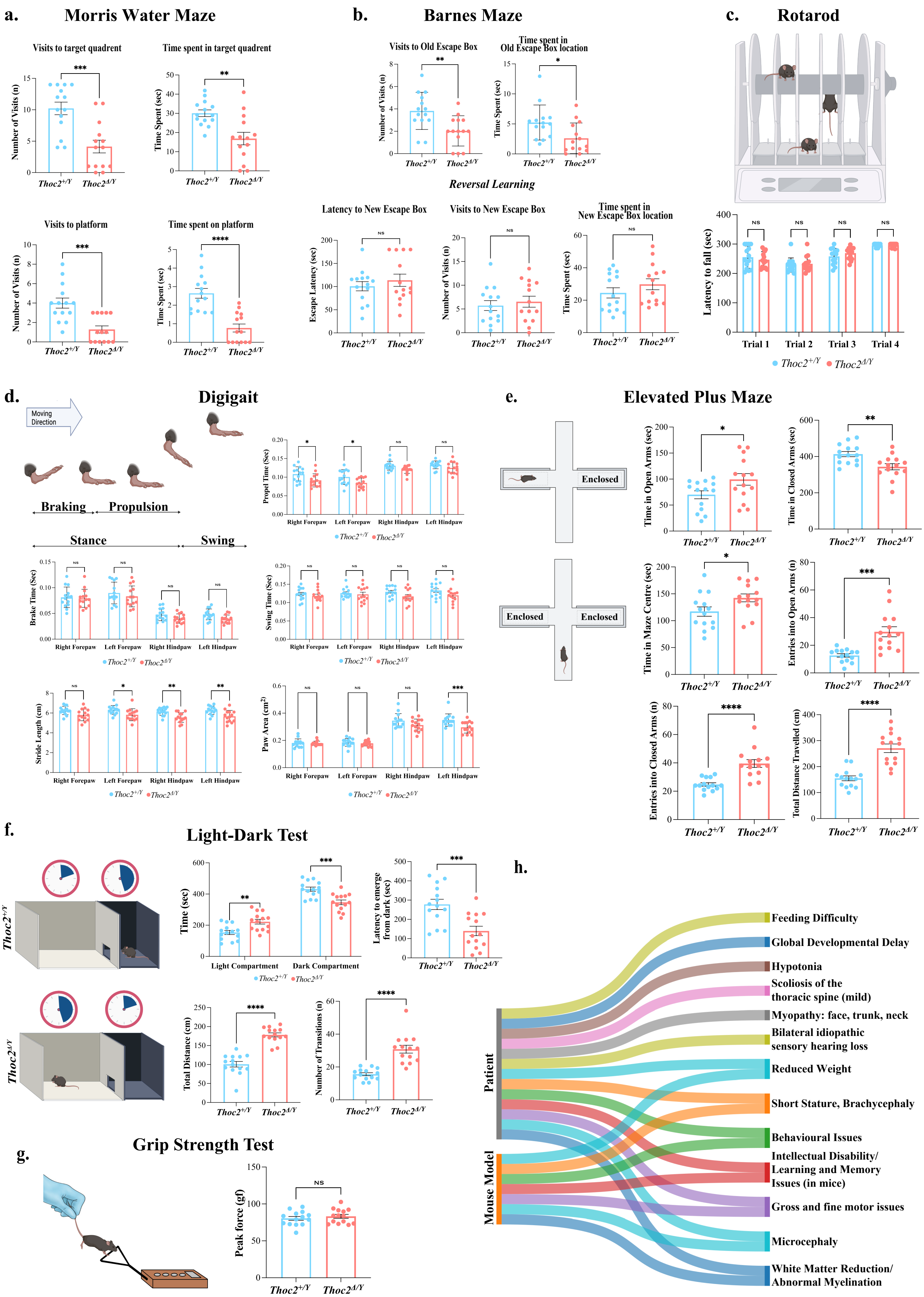

Supplementary Fig. 2: *Thoc2$^{\Delta/Y}$* mice have multiple neurobehavioral deficits. a, Morris Water Maze test graphs showing number of visits to the target quadrant (upper-left) and time spent in the target quadrant (upper-right) and visits to the platform (lower-left) and time spent in the platform (lower-right). **p < 0.01; ***p < 0.001; and ****p < 0.0001; two-tailed unpaired student's t-test. **b,** Barnes Maze test graphs showing number of visits to the old escape box (upper-left) and time spent in the old escape box location (upper-right). Lower reversal learning graphs showing latency to escape into the new escape box on the probe day (lower-left), visits to the new escape box (lower-middle) and time spent in the new escape box location (lower-right). *p < 0.05; **p < 0.01; ns = non-significant; two-tailed unpaired student's t-test. **c,** Rotarod test for assessing the motor coordination. ns = non-significant; unpaired student's t-test. **d,** Gait analysis using the Digigait platform. Graphs showing gait parameters, for each paw/limb of the animal, such as propel, brake, swing, length of stride and paw area. ns = non-significant, *p < 0.05; **p< 0.01; ***p< 0.001; two-way ANOVA, Bonferroni's multiple comparison test. **e,** Elevated Plus Maze test for assessing anxiety like behavior. Graphs showing entries into different arms of the maze, amount of time spent in the arms and the total distance travelled by the animals. *p < 0.05; **p < 0.01; ***p< 0.001; ****p < 0.0001; ; two-tailed unpaired student's t-test. **f,** Light-Dark test for assessing anxiety like behavior. Graphs showing time spent in the light and dark compartments, latency to emerge from the dark, number of transitions between light-dark zones and the total distance travelled by the animals. **p < 0.01; ***p< 0.001; ****p< 0.0001; ; two-tailed unpaired student's t-test (latency to emerge from dark, number of transitions and total distance travelled); two-way ANOVA, Bonferroni's multiple comparison test (time spent in light and dark compartments). **g,** Grip strength analysis using digital grip strength meter**.** ns = non-significant; ; two-tailed unpaired student's t-test. All tests were performed with n = 14 mice per genotype and the data presented are as mean values ± SEM. **g,** Sankey Plot showing overlapping phenotypes between *Thoc2$^{\Delta/Y}$* mice and the *THOC2* exon 37-38 deletion patient. Source data are provided as a Source Data file. Schematics in figures c-g were created with BioRender.com.

Supplementary Fig. 3

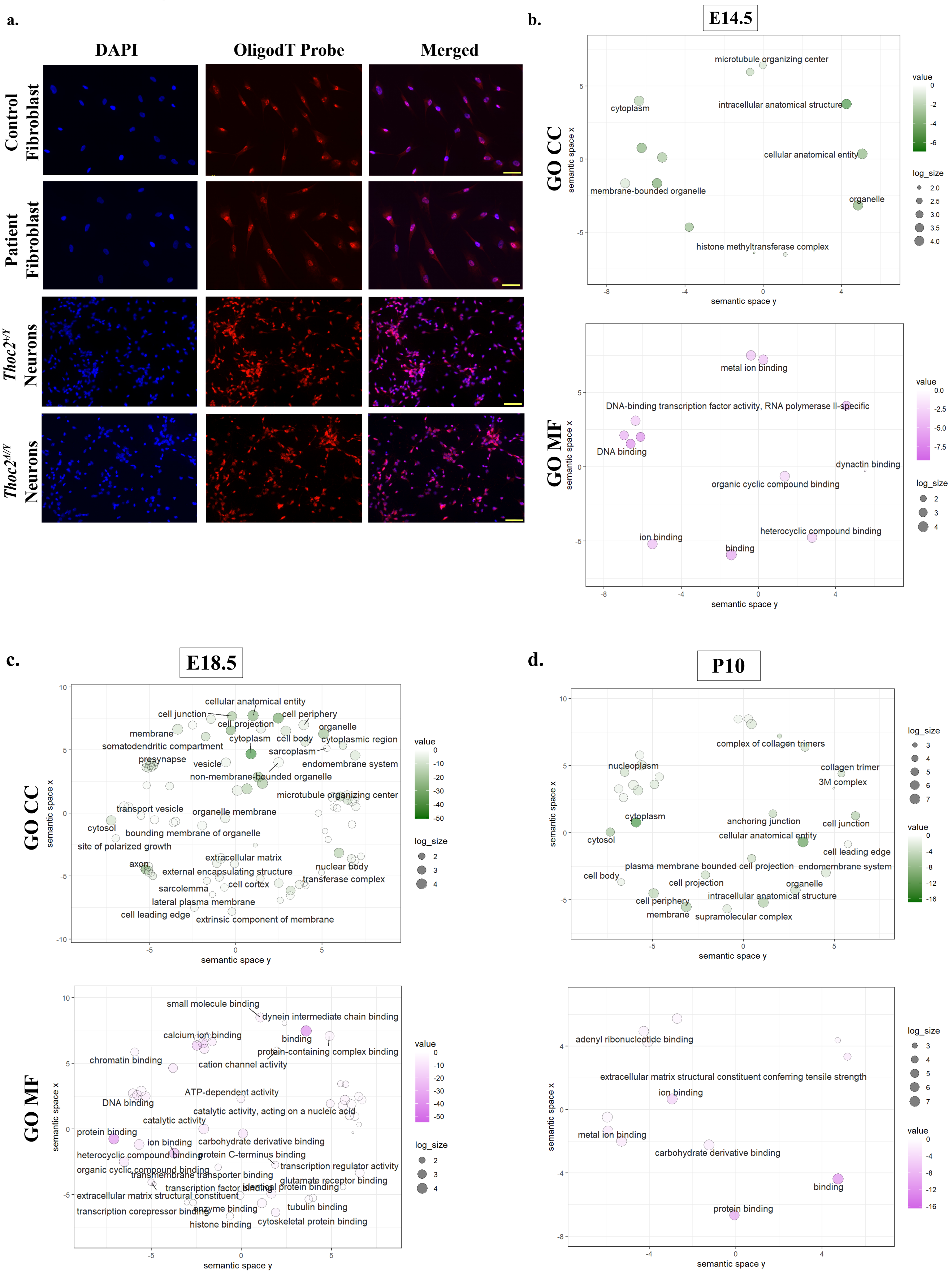

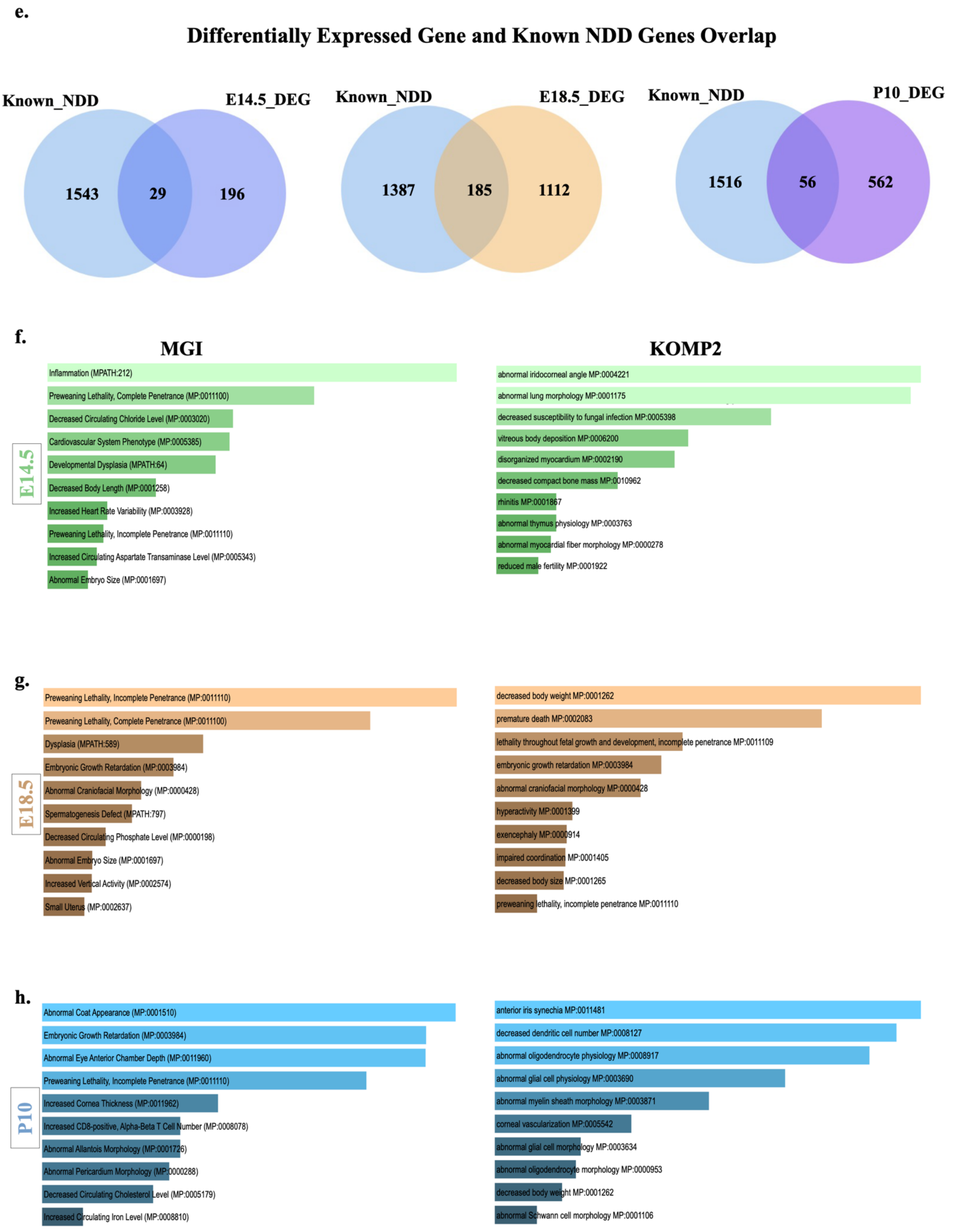
e.
Differentially Expressed Gene and Known NDD Genes Overlap
Known_NDD
E14.5_DEG
1543
29
196
Known_NDD
E18.5_DEG
1387
185
1112
Known_NDD
P10_DEG
1516
56
562
f.
MGI
KOMP2
E14.5
Inflammation (MPATH:212)
Preweaning Lethality, Complete Penetrance (MP:0011100)
Decreased Circulating Chloride Level (MP:0003020)
Cardiovascular System Phenotype (MP:0005385)
Developmental Dysplasia (MPATH:64)
Decreased Body Length (MP:0001258)
Increased Heart Rate Variability (MP:0003928)
Preweaning Lethality, Incomplete Penetrance (MP:0011110)
Increased Circulating Aspartate Transaminase Level (MP:0005343)
Abnormal Embryo Size (MP:0001697)
abnormal iridocorneal angle MP:0004221
abnormal lung morphology MP:0001175
decreased susceptibility to fungal infection MP:0005398
vitreous body deposition MP:0006200
disorganized myocardium MP:0002190
decreased compact bone mass MP:0010962
rhinitis MP:0001867
abnormal thymus physiology MP:0003763
abnormal myocardial fiber morphology MP:0000278
reduced male fertility MP:0001922
g.
E18.5
Preweaning Lethality, Incomplete Penetrance (MP:0011110)
Preweaning Lethality, Complete Penetrance (MP:0011100)
Dysplasia (MPATH:589)
Embryonic Growth Retardation (MP:0003984)
Abnormal Craniofacial Morphology (MP:0000428)
Spermatogenesis Defect (MPATH:797)
Decreased Circulating Phosphate Level (MP:0000198)
Abnormal Embryo Size (MP:0001697)
Increased Vertical Activity (MP:0002574)
Small Uterus (MP:0002637)
decreased body weight MP:0001262
premature death MP:0002083
lethality throughout fetal growth and development, incomplete penetrance MP:0011109
embryonic growth retardation MP:0003984
abnormal craniofacial morphology MP:0000428
hyperactivity MP:0001399
exencephaly MP:0000914
impaired coordination MP:0001405
decreased body size MP:0001265
preweaning lethality, incomplete penetrance MP:0011110
h.
P10
Abnormal Coat Appearance (MP:0001510)
Embryonic Growth Retardation (MP:0003984)
Abnormal Eye Anterior Chamber Depth (MP:0011960)
Preweaning Lethality, Incomplete Penetrance (MP:0011110)
Increased Cornea Thickness (MP:0011962)
Increased CD8-positive, Alpha-Beta T Cell Number (MP:0008078)
Abnormal Allantois Morphology (MP:0001726)
Abnormal Pericardium Morphology (MP:0000288)
Decreased Circulating Cholesterol Level (MP:0005179)
Increased Circulating Iron Level (MP:0008810)
anterior iris synechia MP:0011481
decreased dendritic cell number MP:0008127
abnormal oligodendrocyte physiology MP:0008917
abnormal glial cell physiology MP:0003690
abnormal myelin sheath morphology MP:0003871
corneal vascularization MP:0005542
abnormal glial cell morphology MP:0003634
abnormal oligodendrocyte morphology MP:0000953
decreased body weight MP:0001262
abnormal Schwann cell morphology MP:0001106

Supplementary Fig. 3: *Thoc2$^{\Delta/Y}$* embryonic and adult mice brains show significantly dysregulated transcriptome. a, Representative images of polyA+ FISH performed in control fibroblast, *THOC2* exon 37-38 deletion patient fibroblast, *Thoc2$^{+/Y}$* and *Thoc2$^{\Delta/Y}$* primary neurons at DIV14 of culture. GO terms for CC and MFs enrichment shown as ReviGO plots for significantly dysregulated genes in E14.5 (**b**), E18.5 (**c**), and P10 (**d**) *Thoc2$^{\Delta/Y}$* mice brains. **e,** Venn diagrams showing overlap of significantly dysregulated genes among the E14.5, E18.5 and P10 *Thoc2$^{\Delta/Y}$* brains and known NDD genes. Mouse phenotype ontological enrichment analysis of significantly dysregulated genes in E14.5 (**f**), E18.5 (**g**), and P10 (**h**) *Thoc2$^{\Delta/Y}$* brain. GO, Gene Ontology; CC, Cellular Component; MF, Molecular Function; MGI, Mouse Genome Informatics; KOMP2, Knockout Mouse Phenotyping Program 2. Source data are provided as a Source Data file.

Supplementary Fig. 4

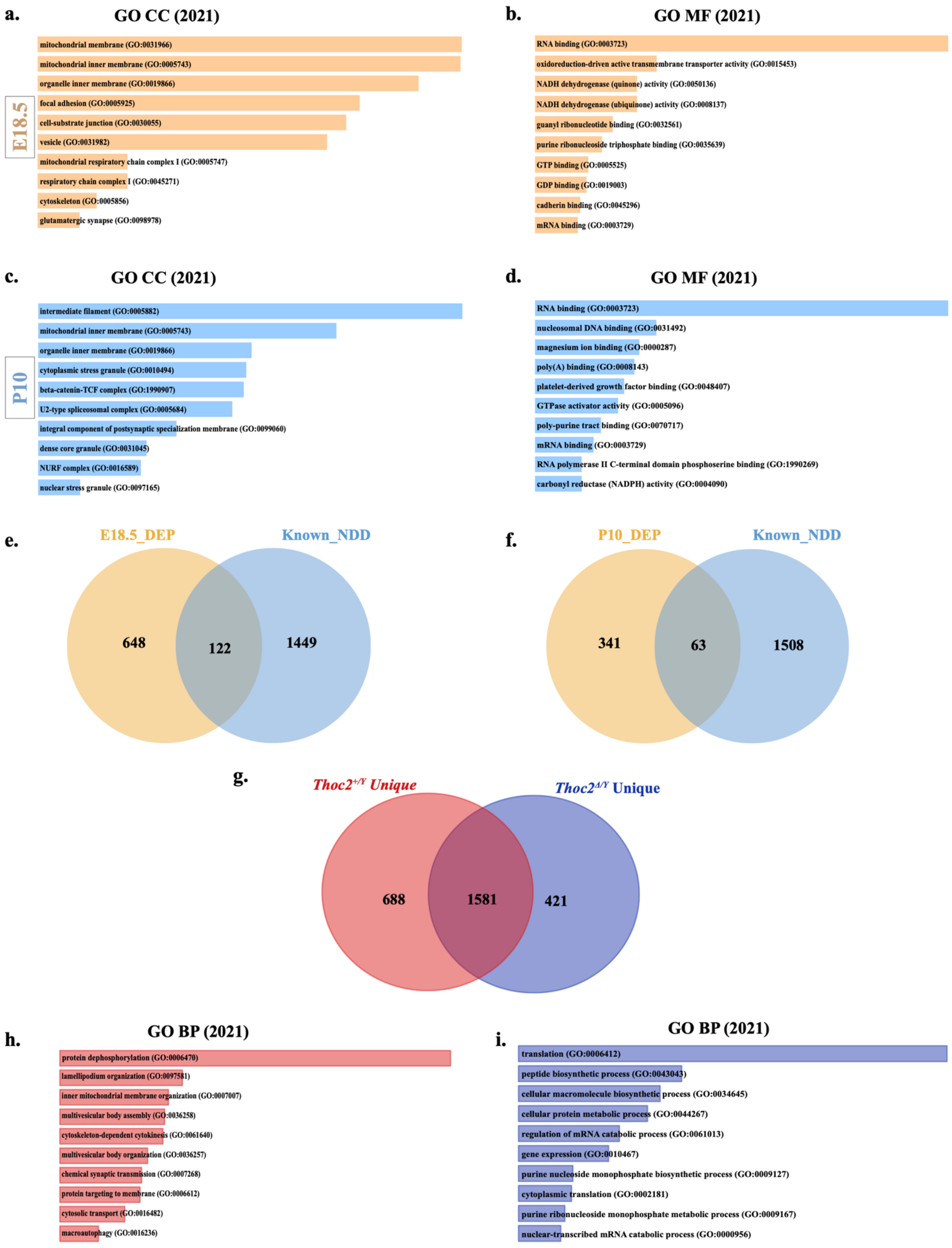

Supplementary Fig. 4: *Thoc2$^{\Delta/Y}$* embryonic and adult mice brains show significantly dysregulated proteome. GO enrichment analyses plots of significantly dysregulated proteins in E18.5 (a-b) and P10 (c-d) *Thoc2$^{\Delta/Y}$* mice brains performed using Enrichr web-tool. Venn diagrams showing overlap of significantly dysregulated proteins between E18.5 (e) and P10 (f) *Thoc2$^{\Delta/Y}$* mice brains and known NDD genes. g, Venn diagrams showing common and unique proteins that change over E18.5 to P10 stage in *Thoc2$^{+/Y}$* and *Thoc2$^{\Delta/Y}$* mouse brain. GO enrichment analyses plots of uniquely changed proteins in *Thoc2$^{+/Y}$* (h) and *Thoc2$^{\Delta/Y}$* (i) mice brains from E18.5 to P10 stage. For a-d and h-i: The top 10 enriched GO terms are shown. The length of the horizontal axis represents the enrichment p-value for the functional clustering. DEP, Differentially Expressed Proteins; GO, Gene Ontology; BP, Biological Process; CC, Cellular Component; MF, Molecular Function. Source data are provided as a Source Data file.

Supplementary Fig. 5

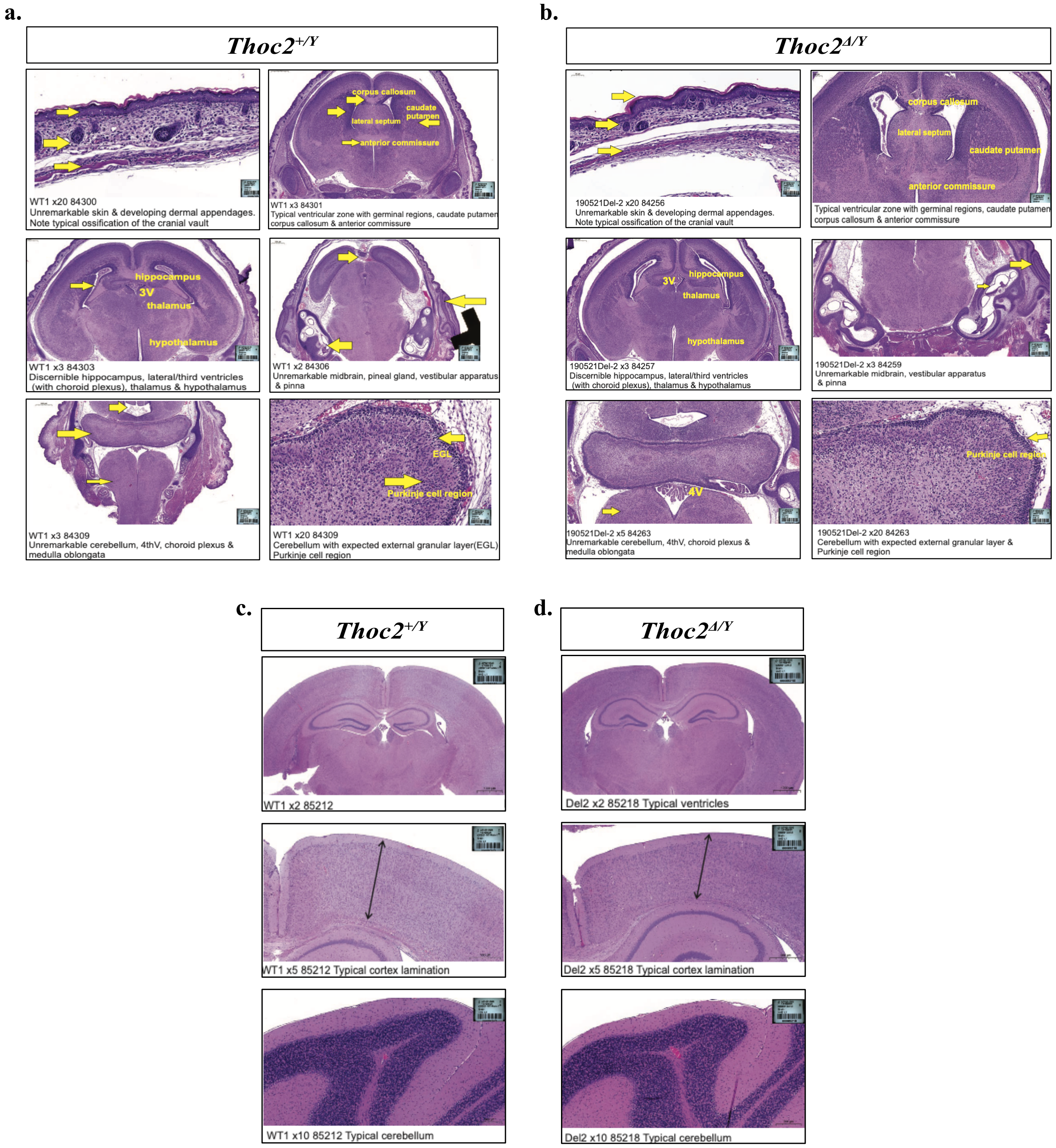

Supplementary Fig. 5: *Thoc2*$^{\Delta/Y}$ embryonic and adult mice do not show any gross morphological alteration in their brain. a-b, Representative images of H&E staining of E18.5 embryonic brain coronal sections. Different histological landmarks were annotated within the pictures and compared between *Thoc2*$^{+/Y}$ and *Thoc2*$^{\Delta/Y}$ mice. (n =4 embryos per genotype). c-d, Representative images of H&E staining of 30 days old mice brain coronal sections. Different histological landmarks were compared between *Thoc2*$^{+/Y}$ and *Thoc2*$^{\Delta/Y}$. (n = 4 mice per genotype).

Supplementary Fig. 6

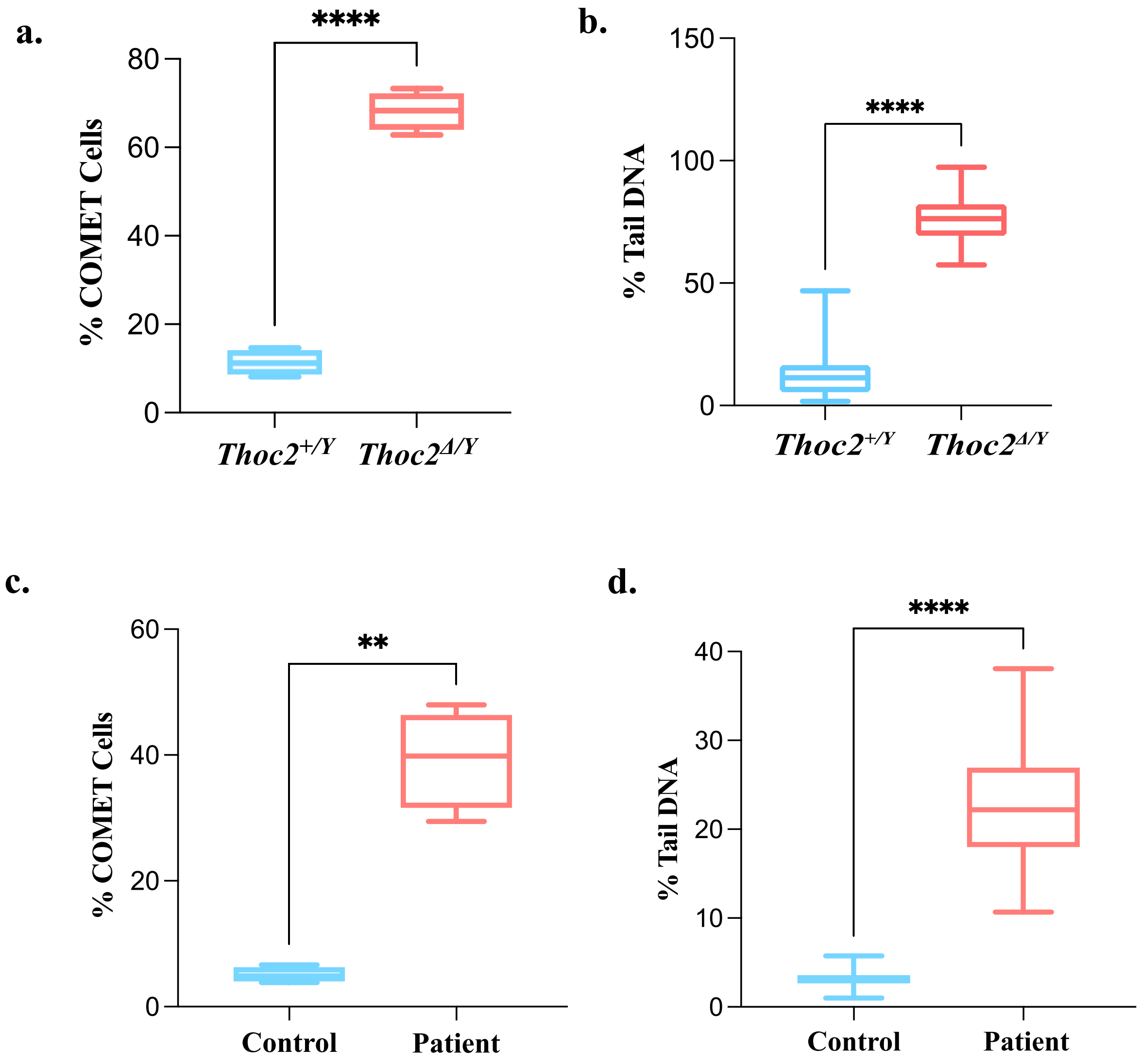

Supplementary Fig. 6: *Thoc2$^{Δ/Y}$* mouse NSCs and *THOC2* patient fibroblasts show DNA damage. Box plots showing **a,** percentage of cells with distinct comet *Thoc2$^{+/Y}$* and *Thoc2$^{Δ/Y}$* NSCs and **b,** percentage of DNA in comet tails of NSCs. n = 4 embryos per genotype and experiments performed in technical triplicates; ****$p < 0.0001$; two-tailed unpaired student's t-test. Box plots showing **c,** percentage of cells with distinct comet tail in control and *THOC2* exon 37-38 deletion patient fibroblasts. and **d,** percentage of DNA in comet tails of the cells. Data presented as mean values ± SEM (n = 3 independent experiments; individual experiment performed in technical quadruplicates); **$p < 0.01$; ****$p < 0.0001$; two-tailed unpaired student's t-test. In all box plots, the boundary of the box closest to zero indicates the 25th percentile and the boundary farthest from zero indicates the 75th percentile. A line within the box shows the median, and the whiskers above and below the box indicate the maximum and minimum points of the dataset, respectively. Source data are provided as a Source Data file.

Supplementary Fig. 7

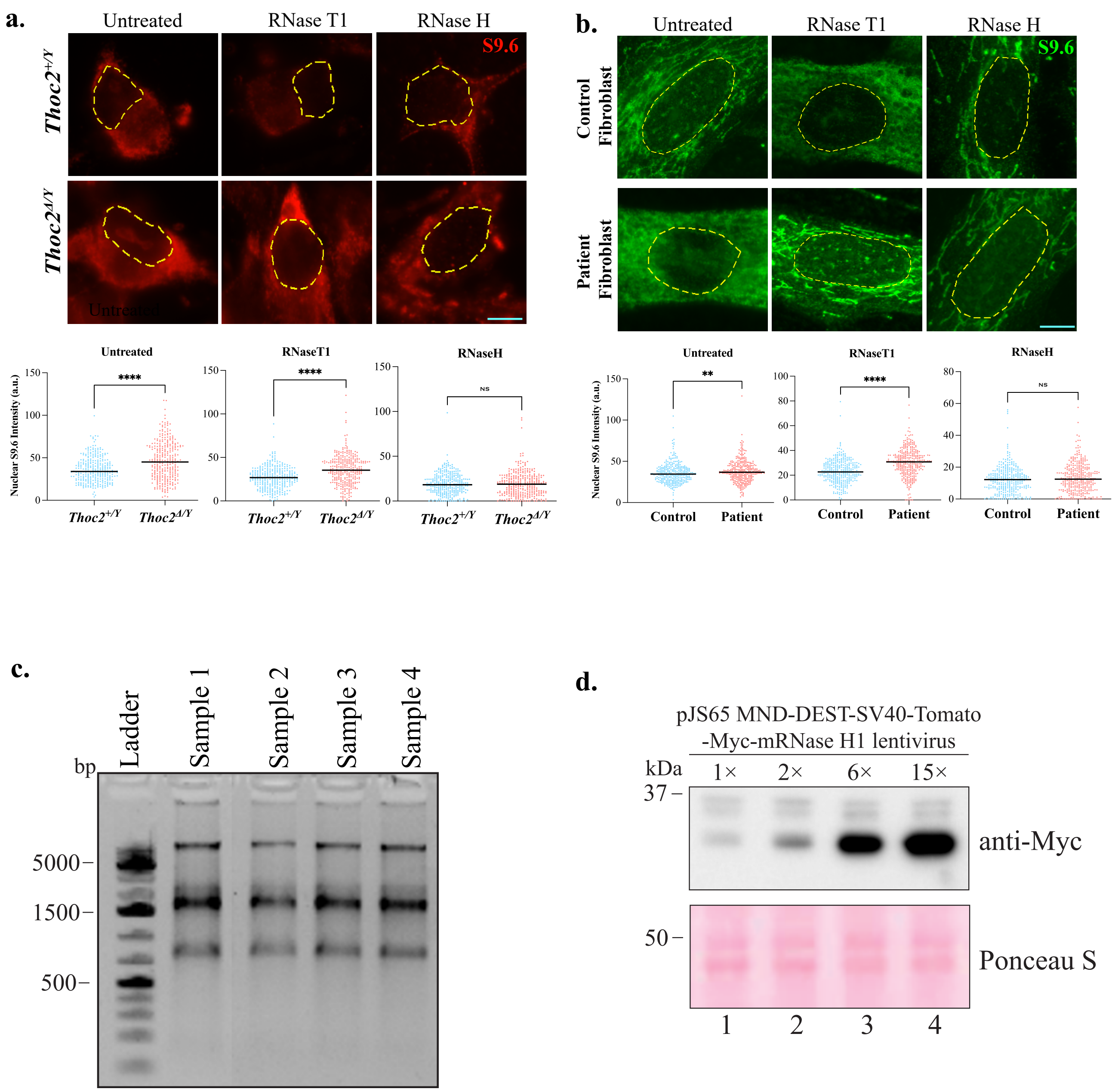

Sample 1: *Thoc2*$^{+/Y}$ NSC
Sample 2: *Thoc2*$^{\Delta/Y}$ NSC
Sample 3: *THOC2* Ex37-38Del Patient Fibroblast
Sample 4: Control Fibroblast

Supplementary Fig. 7: Increased R-loops in *Thoc2*$^{\Delta/Y}$ NSCs and *THOC2* ex37- 38 deletion patient fibroblasts

Representative immunofluorescent images of untreated, RNase T1 or RNase H treated *Thoc2*$^{+/Y}$ and *Thoc2*$^{\Delta/Y}$ NSCs (**a**) and control and *THOC2* exon 37-38 deletion patient fibroblasts (**b**) stained with anti-RNA:DNA Hybrid S9.6 antibody (Kerafast, AB01137-23.0). Scatter plots showing quantitative measurement of mean nuclear S9.6 fluorescence intensity using ImageJ software (n = 3 independent experiments); $^{**}p < 0.01$; $^{****}p < 0.0001$; *Mann–Whitney U test with the black line indicating median value*. a.u, arbitrary units. **c,** Representative agarose gel image showing equal amounts of gDNA used in the DOT Blot assay. 2µg gDNA of each sample was resolved on a 1% Agarose Gel (in 1× TBE buffer). The gel shows amount of DNA loaded in each well with ribosomal RNA bands also visible. **d,** Western blot showing expression of RNase H1 in HEK293T cells transduced with different MOIs of RNase H1 lentivirus. The Ponceau-S-stained image of the blot at bottom panel shows equal protein loading. Source data are provided as a Source Data file.

Supplementary Fig. 8

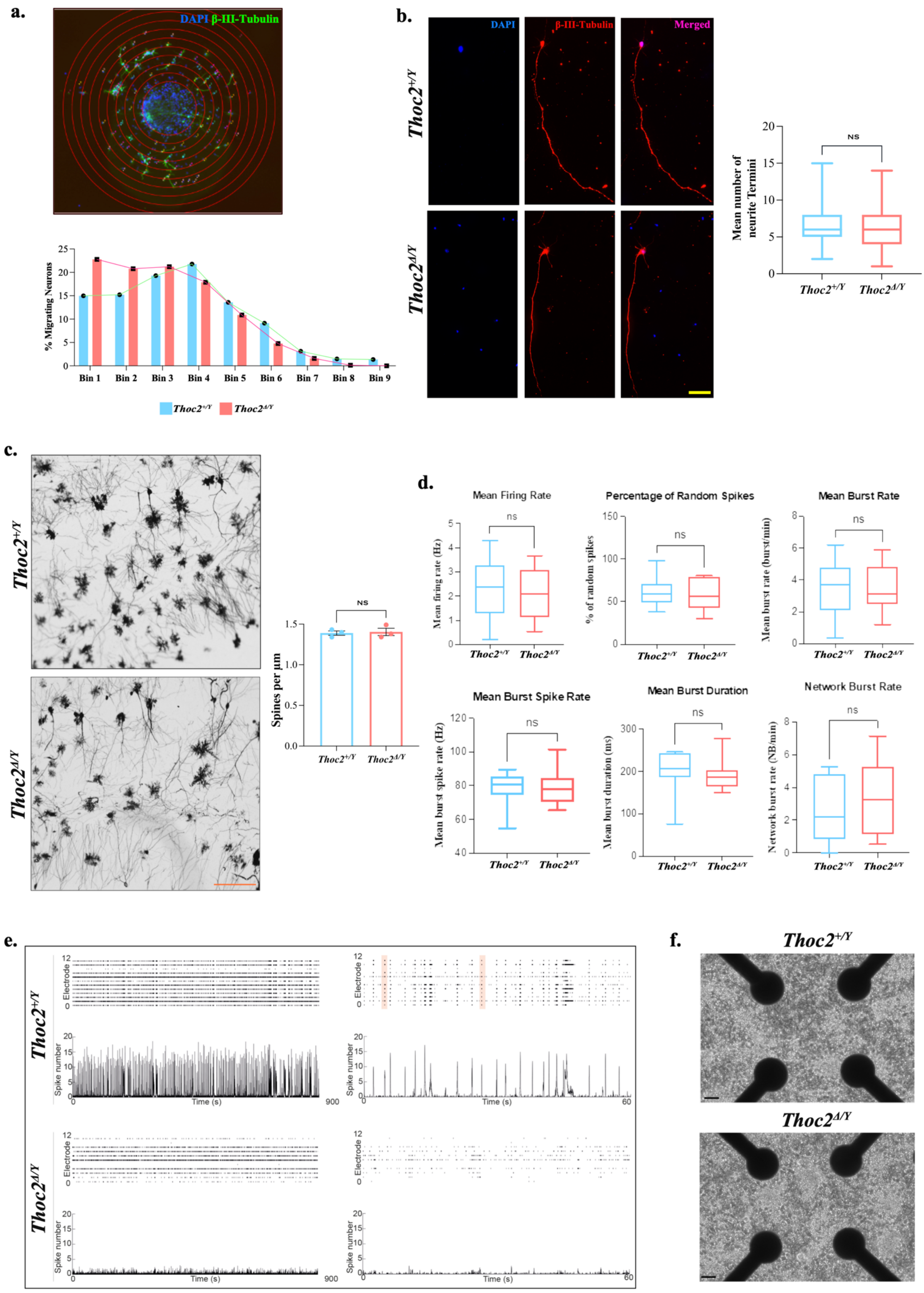

Supplementary Fig. 8: Structural and functional assessment of *Thoc2*$^{Δ/Y}$ mice neurons. a, Representative immunofluorescent image of neuronal migration assay showing the concentric circle approach used for measurement of neural migration away from seeded *Thoc2*$^{+/Y}$ neurosphere (see Fig. 8c). Graph showing Percentage of neurons within each bin and the trend of migration with increasing bin size. Data presented as mean values (each bin is 45µm). Also see Fig. 8b for data output. **b,** Representative immunofluorescent images of *Thoc2*$^{+/Y}$ and *Thoc2*$^{Δ/Y}$ primary neurons at day *in vitro* 5 in cultures. Scale Bar: 20µm. Neurons were stained with ß-III Tubulin marker (red). Box plots showing quantification of the neurite termini. n = 4 embryos per genotype; independent experiments performed in technical triplicate; ns = non-significant; unpaired student's t-test. The boundary of the box closest to zero indicates the 25th percentile and the boundary farthest from zero indicates the 75th percentile. A line within the box shows the median, and the whiskers above and below the box indicate the maximum and minimum points of the dataset, respectively. **c,** Left Panel: Representative low magnification images of Golgi-Cox stained *Thoc2*$^{+/Y}$ *and Thoc2*$^{Δ/Y}$ mouse brains showing pyramidal neurons. Scale Bar: 200µm. Right Panel: Graph showing quantification of spine density per µm length of a dendrite. Data presented as mean values ± SEM (n = 3 mice per genotype; Total 1852 spines for *Thoc2*$^{+/Y}$ and 2055 spines for *Thoc2*$^{Δ/Y}$ mice were analysed); ns: not significant; students t-test. **d,** Quantitative measurement of different neural network parameters at immature stage of neuron culture (DIV7). Data presented as mean values ± SEM (n = 4 embryos per genotype); ns = non-significant; Kolmogorov-Smirnov test. **e,** Representative raster plots and spike time histogram of *Thoc2*$^{+/Y}$ (upper panel) and *Thoc2*$^{Δ/Y}$ (lower panel) neuronal networks at DIV14. The histogram and raster plot depict the whole 900 seconds recording (left panel) and a 60 second zoom-in (right panel), displaying synchronous network bursts (in orange highlight) in the *Thoc2*$^{+/Y}$ neuronal networks. **f,** Representative images showing plating density of *Thoc2*$^{+/Y}$ *and Thoc2*$^{Δ/Y}$ neurons in the MEA plate. Source data are provided as a Source Data file.

Supplementary Fig. 9

a.

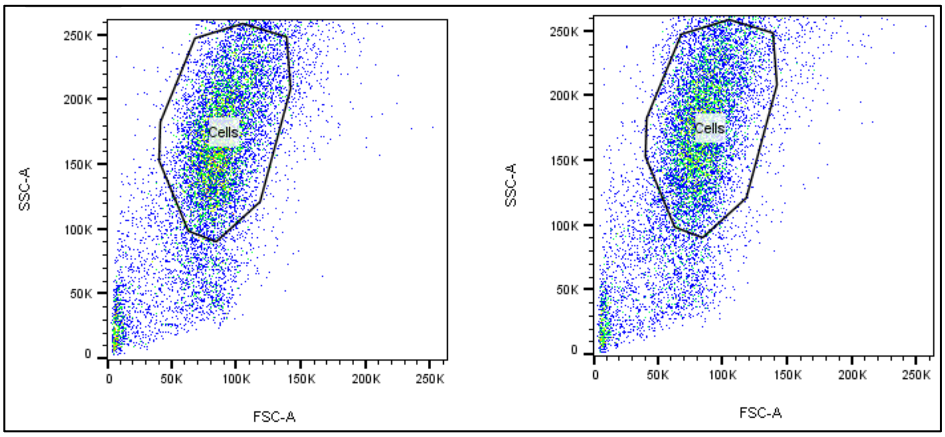

b.

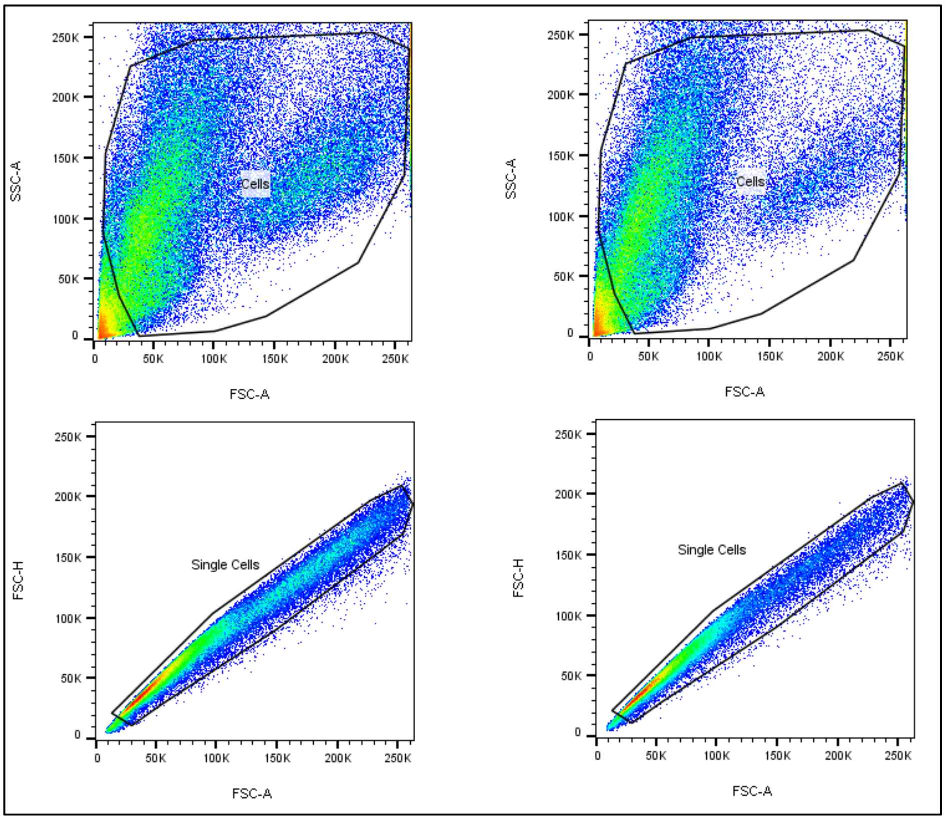

Supplementary Fig. 9: Gating Strategy used for analysing Flow Cytometry data. **a,** Forward Scatter Area (FSC-A) on the x-axis and Side Scatter Area (SSC-A) on the y-axis were used to gate the Neural Stem Cells (NSC) for analysing the cell cycle stage using Hoechst staining (**Fig. 6a**). **b,** FSC-A and SSC-A were first used to identify NSC population and then single cells were gated using FSC-A on the x-axis and Forward Scatter Height (FSC-H) on the y-axis to analyse Annexin-V-stained apoptotic cells (**Fig. 6c**).
