# Supplementary material for: Compromised transcription-mRNA export factor THOC2 causes R-loop accumulation, DNA damage and adverse neurodevelopment
Source: Nat Commun. 2024 Feb 8;15:1210. doi: 10.1038/s41467-024-45121-5 (PMC10853216; doi:10.1038/s41467-024-45121-5)
Supplement: Supplementary file 3 — Description of Additional Supplementary Files [file 41467_2024_45121_MOESM3_ESM.pdf]

Description of Additional Supplementary Files

File Name: Supplementary Data 1
Description: Mouse CRISPR trial details

File Name: Supplementary Data 2
Description: Patient clinical features

File Name: Supplementary Data 3
Description: RNA Seq analysis data

File Name: Supplementary Data 4
Description: Proteomics analysis data

File Name: Supplementary Data 5
Description: PCR primers, CRISPR oligos and PCR conditions

File Name: Supplementary Data 6
Description: Antibody details
