# Supplementary material for: Compromised transcription-mRNA export factor THOC2 causes R-loop accumulation, DNA damage and adverse neurodevelopment
Source: Nat Commun. 2024 Feb 8;15:1210. doi: 10.1038/s41467-024-45121-5 (PMC10853216; doi:10.1038/s41467-024-45121-5)
Supplement: Supplementary file 10 — Reporting Summary [file 41467_2024_45121_MOESM10_ESM.pdf]

nature portfolio

Corresponding author(s): Professor Jozef Gecz

Last updated by author(s): Jan 8, 2024

Reporting Summary

Nature Portfolio wishes to improve the reproducibility of the work that we publish. This form provides structure for consistency and transparency in reporting. For further information on Nature Portfolio policies, see our Editorial Policies and the Editorial Policy Checklist.

Statistics

For all statistical analyses, confirm that the following items are present in the figure legend, table legend, main text, or Methods section.

n/a	Confirmed	
☐	☒	The exact sample size (*n*) for each experimental group/condition, given as a discrete number and unit of measurement
☐	☒	A statement on whether measurements were taken from distinct samples or whether the same sample was measured repeatedly
☐	☒	The statistical test(s) used AND whether they are one- or two-sided<br>*Only common tests should be described solely by name; describe more complex techniques in the Methods section.*
☐	☒	A description of all covariates tested
☐	☒	A description of any assumptions or corrections, such as tests of normality and adjustment for multiple comparisons
☐	☒	A full description of the statistical parameters including central tendency (e.g. means) or other basic estimates (e.g. regression coefficient) AND variation (e.g. standard deviation) or associated estimates of uncertainty (e.g. confidence intervals)
☐	☒	For null hypothesis testing, the test statistic (e.g. *F*, *t*, *r*) with confidence intervals, effect sizes, degrees of freedom and *P* value noted<br>*Give P values as exact values whenever suitable.*
☒	☐	For Bayesian analysis, information on the choice of priors and Markov chain Monte Carlo settings
☒	☐	For hierarchical and complex designs, identification of the appropriate level for tests and full reporting of outcomes
☒	☐	Estimates of effect sizes (e.g. Cohen's *d*, Pearson's *r*), indicating how they were calculated

Our web collection on statistics for biologists contains articles on many of the points above.

Software and code

Policy information about availability of computer code

Data collection	H&E Stained images were acquired using 3D Histech Pannoramic Scan II machine. Golgi-Cox stained brain sections were imaged using Olympus IX83 microscope (Olympus, Japan). Immunofluorescence stained images were captured using a Zeiss Axioplan2 microscope (Carl Zeiss, Jena, Germany) equipped with an HBO 100 lamp (Carl Zeiss), Axiocam Mrm camera and V.4.9.1.0 software. Electrophysiology data were collected using a 24-well multielectrode arrays (MEAs) machine (Multichannel systems, MCS GmbH, Reutlingen, Germany). Whole genome and RNA sequencing was performed using a Illumina NovaSeq 6000 system. Proteomics data was collected using a nano-liquid chromatography (nLC) on the Dionex RSLC 3500 system. Mouse Behavioural testing data was collected using the CleverSys TopScan Software Suite (v3.0), ANY-maze video tracking system v4.99m, TruScan locomotor system (v1.0).
Data analysis	GraphPad Prism v9 was used for general statistics. Flow cytometry data was analyzed using FlowJo V. 10.0.8. RNA sequemcing data using Salmon v1.6.097, edgeR v 3.40.2 package in the R v 4.2.2 environment. Proteomics data using Spectronaut™ v16.3 and Whole genome sequencing using GATKv4.1.6. Comet assay data was analysed using CaspLab software v1.2. Electrophysiology data analyzed using Multiwell analyzer software (Multichannel systems) and MATLAB (The Mathworks, Natick, MA, USA). Gene ontology analysis was performed using Online ShinyGO v0.76 software.

For manuscripts utilizing custom algorithms or software that are central to the research but not yet described in published literature, software must be made available to editors and reviewers. We strongly encourage code deposition in a community repository (e.g. GitHub). See the Nature Portfolio guidelines for submitting code & software for further information.

Data

Policy information about availability of data

All manuscripts must include a data availability statement. This statement should provide the following information, where applicable:

- Accession codes, unique identifiers, or web links for publicly available datasets
- A description of any restrictions on data availability
- For clinical datasets or third party data, please ensure that the statement adheres to our policy

The whole genome sequencing and RNA-Seq data have been deposited in the Gene Expression Omnibus (GEO) database with project code GSE245539 (https://www.ncbi.nlm.nih.gov/geo/query/acc.cgi?acc=GSE245539). The proteomics data have been deposited in Pride Proteome Exchange repository with accession number PXD040358. All data used for evaluating the conclusions are presented in the main manuscript and/or the Supplementary Materials. Source Data are also provided with this paper.

RNA-seq:
Sequence read pairs from each sample were quantified against mouse transcripts using Salmon v1.6.097 with prebuilt indexes for the mouse mm10 genome build available from RefGenie (http://refgenomes.databio.org/v3/assets/splash/0f10d83b1050c08dd53189986f60970b92a315aa7a16a6f1/salmon_sa_index?tag=default)989. Differential gene and transcript expression between groups was assessed at each developmental time point separately (E14.5, E18.5 and P10) using the default options for the glmQLFit function on transcripts that were included after using the filterByExpr function from the edgeR v 3.40.2 package in the R v 4.2.2 environment99. Lists of expressed genes or transcripts not excluded by the filterByExpr function at each time point were used as the background in subsequent gene-ontology analyses. Gene ontology term enrichments were analysed using the g:GOSt functional profiling tool available from the g:Profiler web service https://biit.cs.ut.ee/gprofiler/gost39. ENSEMBL mouse gene ID were submitted as an ordered query ranked by ascending p-values to a cut off value < 0.01 for each time point. Significantly enriched terms (FDR < 0.05) identified from g:GOSt analysis were then clustered using the REVIGO web service http://revigo.irb.hr/100. The percent GC content of the differentially expressed genes were compared to the mouse genome using ShinyGO v0.76.

Proteomics:
The raw data files from each sample generated on the timsTOF Pro mass spectrometer were processed using the software package Spectronaut™ v16.3 (Biognosys)102. directDIA™ analysis was performed and the data were searched against the Uniprot Mus musculus FASTA database (17,056 entries; Year 2021). The following additional parameters were applied: variable modifications - deamidation (N/Q), oxidation (M); fixed modification – methylthio (C); enzyme – Trypsin/P; missed cleavages – 2. All other parameters were set to default values. Proteins with a false discovery rate (FDR) of ≤ 1% were reported. Gene Ontology (GO) enrichment analysis was performed using Enrichr web-platform103,104. For the Enricher plots, p-value values for function clustering and multiple detection corrections were calculated, and the plots were sorted based on the Benjamini-Hochberg corrected p-value represented as the length of the GO term bars. Volcano plots were generated using R-studio 105. Venn diagrams were generated using jvenn online platform 10607. The percent GC content of the genes corresponding to dysregulated proteins were compared to the mouse genome using ShinyGO v0.76101.

Whole Genome Sequencing:
Sequence reads were mapped to GRCm38 with BWA-MEM (0.7.15) and variants were called using GATKv4.1.6. All variants were annotated for allele frequency, locus identity and likely pathogenicity using SnpEff 94 and filtered for significance using SnpSift95. The copy number variations were detected and characterized using CNVnator96. The alignments for the sequence reads were viewed with the Integrative Genomics Viewer (IGV) v.2.14.0.

Research involving human participants, their data, or biological material

Policy information about studies with human participants or human data. See also policy information about sex, gender (identity/presentation), and sexual orientation and race, ethnicity and racism.

Reporting on sex and gender	sex: Male, dermal fibroblasts generated from skin biopsy.
Reporting on race, ethnicity, or other socially relevant groupings	No race, ethnicity or socially relevant groupings were applied in this study.
Population characteristics	This is not a population study and no human participant was directly involved.
Recruitment	Skin sample was taken from the patient with the THOC2 exon 37-38 deletion variant (and associated intellectual disability) during follow-up check-up with informed consent with the patient details de-identified.
Ethics oversight	The study was approved by the Women's and Children's Hospital Network (WCHN) and the University of Adelaide Human Research Ethics Committees, approval number REC786-07-2023.

Note that full information on the approval of the study protocol must also be provided in the manuscript.

Field-specific reporting

Please select the one below that is the best fit for your research. If you are not sure, read the appropriate sections before making your selection.

☒ Life sciences ☐ Behavioural & social sciences ☐ Ecological, evolutionary & environmental sciences

For a reference copy of the document with all sections, see nature.com/documents/nr-reporting-summary-flat.pdf

Life sciences study design

All studies must disclose on these points even when the disclosure is negative.

Sample size	G Power web-based free software was used for calculating the group sizes for each outcome. All experiments were informed by our (Jolly et al 2013) and others (Sukoff Rizzo et al 2017) published work for the statistical reproducibility and adequateness of sample size. Sample sizes, statistics and interpretations are given in the relevant figures and text.<br><br>References:<br>1. Sukoff Rizzo SJ, Crawley JN. 2017 Behavioral Phenotyping Assays for Genetic Mouse Models of Neurodevelopmental, Neurodegenerative, and Psychiatric Disorders. Annu Rev Anim Biosci. 5:371-389. doi:10.1146/annurev-animal-022516-022754<br>2. Jolly LA, Homan CC, Jacob R, Barry S, Gecz J. 2013 The UPF3B gene, implicated in intellectual disability, autism, ADHD and childhood onset schizophrenia regulates neural progenitor cell behaviour and neuronal outgrowth. Hum Mol Genet. 22(23):4673-87. doi:10.1093/hmg/ddt315
Data exclusions	No data were excluded from analysis.
Replication	All the reproducibility details including the number of independent biological and/or technical replicates and number of independent experiments are included in the relevant figures and text.
Randomization	Covariates were controlled as the mice are inbred, collected as litters prenatally and group housed prior to weaning. The only covariate is genotype.
Blinding	For all the data collection and experimental set-ups, blinding was not possible because of distinct phenotype of the experimental (Thoc2 ex37-38del) male mice compared to the wild type litter-mate male mice and the requirement of setting-up experiments by clearly marking the source of the experimental materials (e.g. tissue, cells). However, post data collection, investigators were blinded wherever possible (e.g. in electro-physiology analysis, immunofluorescence analysis, single-cell electrophoresis).

Reporting for specific materials, systems and methods

We require information from authors about some types of materials, experimental systems and methods used in many studies. Here, indicate whether each material, system or method listed is relevant to your study. If you are not sure if a list item applies to your research, read the appropriate section before selecting a response.

Materials & experimental systems

n/a	Involved in the study	
☐	☒	Antibodies
☒	☐	Eukaryotic cell lines
☒	☐	Palaeontology and archaeology
☐	☒	Animals and other organisms
☒	☐	Clinical data
☒	☐	Dual use research of concern
☒	☐	Plants

Methods

n/a	Involved in the study	
☒	☐	ChIP-seq
☐	☒	Flow cytometry
☒	☐	MRI-based neuroimaging

Antibodies

Antibodies used	Details of all the antibodies with catalogue numbers and other relevant details are provided in Supplementary Data 6.<br><br>Primary Antibodies<br><br>anti-THOC1 Rabbit Bethyl Laboratories A302-839A (1:1000)<br>anti-THOC2-I Rabbit Sigma-Aldrich HPA047921 (1:1000)<br>anti-THOC2-II Rabbit Bethyl Laboratories A303-630A (1:1000)<br>anti-THOC3 Rabbit Bethyl Laboratories A304-870A (1:1000)<br>anti-THOC5 Rabbit Bethyl Laboratories A302-120A (1:1000)<br>anti-THOC6 Mouse Santa Cruz Biotechnology SC390722 (1:1000)<br>anti-THOC7 Rabbit Bethyl Laboratories A305-247A (1:1000)<br>anti-ALYREF Rabbit Bethyl Laboratories A302-892A (1:1000)<br>anti-UAP56 Rabbit abcam ab181061 (1:1000)<br>anti-CBP80 Rabbit Bethyl Laboratories A301-794A (1:1000)<br>anti-CHTOP Rabbit Bethyl Laboratories A303-412A (1:1000)<br>anti-UIF Rabbit Bethyl Laboratories A303-525A (1:1000)<br>anti-ZC3H11A Rabbit Bethyl Laboratories A301-523A (1:1000)<br>anti-ß-Tubulin III Rabbit Sigma-Aldrich T2200 (1:1000)<br>anti-MAP2 Chicken Sigma-Aldrich AB15452 (1:1000)<br>anti-Pax6 Rabbit Biolegend 901301 (1:1000)<br>anti-CNPase Mouse Sigma-Aldrich C5922 (1:1000)<br>anti-GFAP Chicken Invitrogen PA1-10004 (1:1000)

anti-CIP29 Rabbit Invitrogen PA5-21783 (1:1000)
anti-H2AX Rabbit Cell Signaling Technology 2595S (1:1000 for western blot analysis of neural stem cell lysates) and (1:500 for western blot analysis of E14.5 and E18.5 mouse brain lysates)
anti-gamma-H2AX Mouse Sigma-Aldrich 05636 (1:1000 for western blot analysis of neural stem cell lysates) and (1:500 for western blot analysis of E14.5 and E18.5 mouse brain lysates and immunofluorescence staining of mouse primary neuron)
anti-RNA:DNA Hybrid (S9.6) Rabbit Kerafast AB01137-23.0 (1:1000)
anti-TAU1 Mouse Sigma-Aldrich MAB3420 (1:1000)
anti-Synapsin1 Rabbit abcam ab254349 (1:500)
anti-PSD95 Mouse abcam ab2723 (1:500)
anti-ß-Tubulin Rabbit abcam A6046 (1:1000)
anti-ß-Actin Mouse abcam 8226 (1:1000)
anti-RNA:DNA Hybrid (S9.6) Mouse Sigma-Aldrich MABE1095 (1:1000)
anti-c-Myc (9E10)-HRP Mouse Invitrogen MA1-980-HRP (1:2000)

Secondary Antibodies

anti-Rabbit IgG (H+L)/Alexa Fluor 488 Donkey Invitrogen A21206 (1:1500 for immunofluorescence staining of fixed cells) and (1:1000 for immunofluorescence staining of mouse brain sections)
anti-Rabbit IgG (H+L)/Alexa Fluor 555 Donkey Invitrogen A31572 (1:1500 for immunofluorescence staining of fixed cells) and (1:1000 for immunofluorescence staining of mouse brain sections)
anti-Rabbit IgG (H+L)/Alexa Fluor 647 Donkey Invitrogen A31573 (1:1500)
anti-Mouse IgG (H+L)/Alexa Fluor 488 Donkey Invitrogen A21202 (1:1500 for immunofluorescence staining of fixed cells) and (1:1000 for immunofluorescence staining of mouse brain sections)
anti-Mouse IgG (H+L)/Alexa Fluor 555 Donkey Invitrogen A31570 (1:1500 for immunofluorescence staining of fixed cells) and (1:1000 for immunofluorescence staining of mouse brain sections)
anti-Mouse IgG (H+L)/Alexa Fluor 647 Donkey Invitrogen A31571 (1:1500)
anti-Chicken IgY (H+L)/Alexa Fluor 488 Donkey Invitrogen A78948 (1:1500)
anti-Chicken IgY (H+L)/Alexa Fluor 647 Donkey Invitrogen A78952 (1:1500)
anti-Rabbit IgG/HRP Goat Agilent P044801-02 (1:1000)
anti-Mouse IgG/HRP Goat Agilent P044701-02 (1:1000)

Validation

The URL links of the manufacturer for all the antibodies is included in the Supplementary Data 6. The information provided in the specific data sheets indicated that the antibodies were validated by the respective manufacturer.

Animals and other research organisms

Policy information about studies involving animals; ARRIVE guidelines recommended for reporting animal research, and Sex and Gender in Research

Laboratory animals

C57BL/6JArc mice were housed in individually-ventilated cages (IVCs) with 22-24°C temperature and in 40-70% relative humidity environment. The mice were given access to food and water ad libitum with a 12hr light/dark cycle. For experiments, mice at embryonic stages (E) 14.5, E18.5 and post-natal day (P) 0, P10, P30 and P60 were used. Details relating the mice at the embryonic or post-natal stages used for each experiment are provided in the methods, results, and the respective figure legends.

Wild animals

No wild animals were used in this study

Reporting on sex

As X-chromosome THOC2 gene-associated intellectual disability is observed only in males, male mice (control and variant) were used for the experiments and interpretation.

Field-collected samples

No field collected samples were used in the study.

Ethics oversight

All the animal studies were approved by the University of Adelaide Animal Research Ethics Committee, Adelaide, Australia (approval # M-2020-071).

Note that full information on the approval of the study protocol must also be provided in the manuscript.

Flow Cytometry

Plots

Confirm that:

☒ The axis labels state the marker and fluorochrome used (e.g. CD4-FITC).

☒ The axis scales are clearly visible. Include numbers along axes only for bottom left plot of group (a 'group' is an analysis of identical markers).

☒ All plots are contour plots with outliers or pseudocolor plots.

☒ A numerical value for number of cells or percentage (with statistics) is provided.

Methodology

Sample preparation

Mouse neural stem cells (NSC) were isolated from E18.5 mouse brains, cultured in vitro, and harvested as per standard procedures described in the manuscript. The NSCs were then harvested and washed in 1× PBS, and resuspended in 20µl

	annexin V binding buffer (Hank's balanced salt solution with 1% HEPES and 5 mmol/L CaCl2) with 0.075µg/ml annexin V-PE (Stem Cell Technologies, 100-0331) at 100 000 cells/ml. The cells were stained for 20 minutes at 4°C, diluted with 200µl of ice-cold annexin V binding buffer, and analysed immediately.
Instrument	LSRFortessa X20 Flow cytometer (BD Biosciences)
Software	Flow Cytometry data were analysed using FlowJo V. 10.0.8 software (FlowJo, LLC) and standard protocols for cell cycle and annexin v apoptosis assay.
Cell population abundance	The experiment did not use sorted cells.
Gating strategy	The gating strategy is described in the methods and figures, and is mainly based on cell-specific stain and the scatter parameters. A relevant figure (Supplementary Fig. 9) exemplifying the gating strategy has been provided in the supplementary information file.

☒ Tick this box to confirm that a figure exemplifying the gating strategy is provided in the Supplementary Information.
